# The Patent on Antitoxin

**Published:** 1898-09

**Authors:** 


### THE PATENT ON ANTITOXIN.

Some changes should be made in our present patent laws, judging by the granting of a patent right to Professor Behring, of Germany, for the sole and exclusive sale of Antitoxin in the United States. It is a shame upon the American medical profession to have inflicted upon them the enactment of such patent laws. We do not believe that any United States court of justice will allow this law to stand should an appeal to them be made. Are we to allow a foreign commercial house to dictate to us the use of their products alone? As potent an antitoxic serum as can be found is made right here in the United States. Messrs. Parke, Davis & Co. inform us that they will protect, without one cent of cost to the purchaser, those who may continue to purchase the serum made by that firm. A recent editorial in the *Medical Age* discusses this subject with a great deal of clearness:

"The principle which lies at the foundation of the invention of diphtheria antitoxin and that which underlies all serum therapeutics is, that the blood of immune animals can be used in the treatment of others. Behring did not discover this principle, and in its application he was undoubtedly anticipated by the Japanese workers. If to any single man must be ascribed the distinction of being the inventor and discoverer of the beneficent principle of immunization, the honor belongs to the immortal Pasteur.

"The manufacture of antitoxin has been carried out for many years in England, France, Switzerland, Italy, Russia, and Japan, and in these countries no one has had the temerity to attempt to control exclusively its manufacture. In this country it is made by five boards of health and by several manufacturing firms. In this country alone has an attempt been made to monopolize its production, it being admitted that elsewhere the claims of any patentee are inadmissible.

"If Professor Behring admits any merit in the work of his predecessors and contemporaries, his claim to be the exclusive inventor of diphtheria antitoxin is in contravention of all the ethics of a scientist's career. His claim is an offense against common morality. Had Simpson patented chloroform anesthesia, or had Lister patented antiseptic surgery, the world would have had two selfish empirics, and lost two medical heroes. If Behring, by the righteous judgment of mankind, can be adjudged sole and undisputed inventor of antitoxin, he has a place in the Temple of Fame for achieving the most beneficent discovery of modern times. It remains to be seen whether the temptation to be rich will overcome his ambition to be great, and whether for a tinsel crown he will barter a diadem of everlasting renown."

